# Comparison of peripapillary choroidal thickness between healthy subjects and patients with Parkinson’s disease

**DOI:** 10.1371/journal.pone.0177163

**Published:** 2017-05-16

**Authors:** Elena Garcia-Martin, Luis E. Pablo, Maria P. Bambo, Raquel Alarcia, Vicente Polo, Jose M. Larrosa, Elisa Vilades, Beatriz Cameo, Elvira Orduna, Teresa Ramirez, Maria Satue

**Affiliations:** 1 Ophthalmology Department, Miguel Servet University Hospital, Zaragoza, Spain; 2 Aragon Institute for Health Research (IIS Aragón), University of Zaragoza, Zaragoza, Spain; 3 Neurology Department, Miguel Servet University Hospital, Zaragoza, Spain; 4 Anatomic Pathology Department, Lozano Blesa University Hospital, Zaragoza, Spain; Massachusetts Eye & Ear Infirmary, Harvard Medical School, UNITED STATES

## Abstract

**Purpose:**

To study peripapillary choroidal thickness (PPCT) in healthy subjects using swept-source optical coherence tomography (SS-OCT), and to evaluate PPCT differences between Parkinson´s disease (PD) patients, and age- and sex-matched healthy controls.

**Design:**

Case-control study

**Methods:**

80 healthy subjects and 40 PD patients were consecutively recruited in this single institution study. The healthy subjects were divided into two populations: a teaching population (n = 40, used to establish choroidal zones) and a validating population (n = 40, used to compare measurements with PD patients). An optic disc 6.0×6.0 mm three-dimensional scan was obtained using Deep Range Imaging (DRI) OCT Triton. A 26×26 cube-grid centered on the optic disc was generated to automatically measure choroidal thickness. Five concentric choroidal zones were established and used to compare PPCT between healthy and PD patients.

**Results:**

PPCT was significantly thicker in PD patients compared with controls in all four concentric zones evaluated (p≤0.0001). PPCT followed a similar pattern in controls and PD; it was thicker in the temporosuperior region, followed by the superior, temporal, nasal, and inferior regions.

**Conclusion:**

PD patients presented with an increased PPCT in all zones surrounding the optic disc compared with healthy subjects. The peripapillary choroidal tissue showed a concentric pattern, with the thickness increasing with increasing distance from the optic nerve. SS-OCT could be useful for evaluating choroidal thinning in clinical practice.

## Introduction

Parkinson’s disease (PD) is a neurodegenerative process that leads to the selective loss of dopaminergic neurons, mainly in the basal ganglia of the brain. Clinical manifestations include movement alterations as well as non-motor symptoms, such as dementia, depression, and autonomic dysfunction [1]. Neurons and neural circuits outside the basal ganglia can be affected simultaneously or upstream of the substantia nigra [2].

Vision is one of the non-motor systems altered in PD, especially the visual field corresponding to the fovea [3,4]. Recent studies demonstrated retinal thinning in different macular sectors and retinal nerve fiber layers (RNFL) in PD patients compared with healthy subjects, [5,6], and alterations in multifocal electroretinograms [7,8].

Several mechanisms have been proposed for the axonal loss in PD disease, leading to tissue degeneration and ultrastructural changes of the retinal ganglion cells [9], but the changes of the choroidal layer have not been thoroughly evaluated. Mechanobiologic response of tissues [10] and cells [11] depends on the mode of deformation, and the magnitude and temporal profile of the stimulus, as well as the type of tissue or cell and its biologic state. Understanding the particular deformations observed in each tissue and ocular layer in patients with PD might facilitate diagnosis and treatment.

Before the development of OCT, choroidal studies were limited to histopathologic analysis. OCT is a useful tool for choroidal studies; nevertheless, the role of choroidal analysis for ocular pathologies is not yet established. Spectral domain-optical coherence tomography (SD-OCT), mostly with enhanced depth imaging (EDI), has been used to evaluate the macular and peripapillary choroid (mainly in healthy eyes and glaucoma patients) [12,13], but the relation of OCT measurements with changes in the peripapillary choroid remain unclear. Some studies report a reduction in the mean or regional peripapillary choroidal thickness (PPCT) in primary open-angle glaucoma [14–16], but in these studies, choroidal thickness was measured manually using SD-OCT at only a few points and beneath the circumpapillary ring, an area typically used for RNFL evaluation. The automated segmental measurement software used in the present study, however, is better suited for a broader and more objective evaluation of choroidal thickness. Swept-source (SS) OCT, as compared with SD-OCT with EDI, provides better visualization of the choroid [17], more accurately measures the deep tissues, detects the posterior limit of the sclera, [18], and is applicable for evaluating a broader area of the posterior segment.

Based on the improved ability of this new SS-OCT technology to reveal and automatically measure a wide area of the peripapillary choroid, our first objective was to measure the PPCT in a 26×26 cube-grid centered on the optic disc, which is automatically performed by the Deep Range Imaging (DRI)-OCT Triton (Topcon Corporation, Tokyo, Japan), in a sample of healthy subjects to determine the pattern or distribution of PPCT and to establish objective zones with similar choroidal thicknesses. Our second objective was to study the PPCT differences within these zones in a sample of PD patients compared with age- and sex-matched healthy controls. The third objective was to evaluate the relationship between PPCT alterations and PD severity. The main advantage of the present study is that PPCT was evaluated in a wide area of the peripapillary choroid using an automatic and accurate new method.

## Material and methods

### Study population and design

This was a prospective, observational, cross-sectional case-control study. The study included patients with definite PD, and age- and sex-matched healthy controls. Based on our preliminary studies, we calculated the necessary sample size to detect differences in choroidal thickness of at least 20 μm as measured by OCT, applying a two-tailed test with an alpha of 5% and a beta of 10%, and a risk ratio of 0.5. Based on this calculation, at least 70 eyes were needed (35 from PD patients and 35 from healthy controls). A total of 40 eyes of 40 PD patients and 80 eyes of 80 healthy controls were evaluated. PD diagnosis was based on the UK Brain Bank Criteria, which included, in the first stage, bradykinesia and one additional symptom, i.e., rigidity, 4–6 Hz resting tremor, or postural instability [19,20].

Patients with a visual acuity less than 0.1 (Snellen scale), intraocular pressure (IOP) >20 mmHg, optic neuritis antecedent, no transparent ocular media (nuclear color/opalescence, cortical or posterior subcapsular lens opacity ≥1 according to the Lens Opacities Classification System III system) [21] and systemic disease that could affect the eye (e.g., diabetes, neurologic pathologies, hypertension, and endocrine disorders) were excluded from the study. Subjects with refractive errors greater than 5 diopters (D) of spherical equivalent refraction or 3 D of astigmatism were also excluded from the study.

### Standard protocol approvals, registrations, and patient consent

The study procedures were performed in accordance with the tenets of the Declaration of Helsinki, and the study protocol was reviewed and approved by the Aragon Ethics Committee For Clinical Research before the study began. Written informed consent to participate in the study was obtained from all subjects.

### Main outcome measures

All subjects underwent a complete neuro-ophthalmic examination, including assessment of best-corrected visual acuity using the Snellen chart, pupillary reflexes, and ocular motility; examination of the anterior segment, IOP with the Goldmann applanation tonometer, and papillary morphology by funduscopic exam; as well as OCT.

In the PD group, disease severity was assessed using the Unified Parkinson Disease Rating (UPDRS) and the Hoehn and Yahr scales, and disease duration since the PD diagnosis was recorded. The Hoehn and Yahr scale is a commonly used diagnostic tool for quantifying the progression of PD symptoms [22]. Stages range from 0 (no signs of disease) to 5 (requiring a wheelchair, or bedridden unless assisted). Clinicians and researchers most commonly use the UPDRS, and the motor section in particular, to follow the longitudinal course of PD in clinical studies [23]. The scale includes three sections that evaluate the key areas of disability, and a fourth section that evaluates treatment complications. Treatment for PD was registered using three different categories for clearer classification: “drugs that enhance dopamine levels” (carbidopa, levodopa, and rasagiline), “dopaminergic drugs” (pramipexole, ropirinole, rotigotine), and “other” (amitriptyline, propranolol, clonazepam).

### OCT

An optic disc 6.0×6.0 mm three-dimensional scan was obtained using the DRI OCT Triton (Topcon Corporation). This scan combines morphometric optic disc parameters and various peripapillary parameters, including RNFL and choroidal thickness. The subjects were seated and properly positioned. All DRI-OCT images were obtained by a single well-trained technician blinded to the presence or absence of PD. The DRI-OCT Triton includes the new SMARTTrack tool that enhances tracking, corrects for motion, and guides the operator to reduce potential errors while acquiring images. Only eyes with good quality scans were included in the analysis. Good-quality SS-OCT images were defined as those with a signal strength ≥40 (maximum = 100), and without motion artifact, involuntary saccade, or overt misalignment of decentration. A total of three eyes (two in the PD group and one in the control group) were excluded due to poor DRI-OCT image quality. These eyes were substituted with two new patients in the PD group and one new healthy subject in the control group. The same investigator performed all of the OCT scans and checked the accuracy of segmentation in each scan and the lack of artifacts. A total of 15 scans in the PD group and 10 in the control group were excluded and repeated.

**Fig 1:**
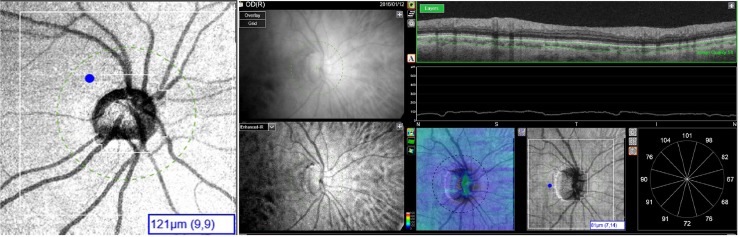
Left: Image of 26×26 cube-grid centered on the optic disc generated to automatically measure choroidal thickness with Deep Range Imaging (DRI) optical coherence tomography (OCT) Triton (Topcon Corporation, Tokyo, Japan). This grid includes 676 cubes (200 μm x 200 μm) around the optic nerve head, but the 88 central cubes corresponding to the optic nerve head area were not analyzed; therefore the DRI-OCT Triton displayed a choroidal thickness for a total of 588 peripapillary cubes. The selected cube marked in the example of Fig 1B corresponds with row number 9 and file number 9. Right: The Bruch membrane and choroidal-scleral interface were delineated with the segmentation algorithm implemented by Topcon.

A 26×26 cube-grid centered on the optic disc was generated to automatically measure choroidal thickness. This grid comprised 676 cubes (200 μm x 200 μm) around the optic nerve head with the 88 central cubes corresponding to the optic nerve head area not analyzed; therefore the DRI-OCT Triton displays choroidal thickness for a total of 588 peripapillary cubes (Fig 1). The Bruch membrane and choroidal-scleral interface were delineated with the segmentation algorithm implemented by Topcon (Fig 1).

### A total of 120 eyes were analyzed

80 healthy controls and 40 PD patients. The healthy control group was randomly divided in two populations: the teaching population (n = 40 controls, used to establish choroidal zones) and the validating population (n = 40 controls, used to compare measurements with PD patients). Sex and age were not significantly different between the control groups or between each of the control groups and the PD group. Only right eyes were selected for the statistical analysis, because choroidal thickness is reported to differ between right and left eyes [23,24].

### Statistical analysis

Statistical analyses were performed using the Statistical Package for the Social Sciences (SPSS 20.0, SPSS Inc., Chicago, IL). The Kolmogorov-Smirnov test was used to assess the sample distribution of the variables. For quantitative data following a parametric distribution, differences between evaluation groups were compared using the Student´s t-test. For qualitative data, a chi square test was used for comparison. We delimited four areas based on the range of choroidal thicknesses and compared the average of these areas between the PD and control groups. We also compared choroidal thicknesses and volume in the four quadrants and six sectors provided by Triton OCT between PD patients and healthy controls. To calculate volume, we computed the thickness of the four quadrants and the six sectors, and total thickness relative to the effective imaging area; i.e., the total imaging area (peripapillary constant area, 27.04 mm^2^) minus the optic nerve head area (variable area, 3.52 mm^2^). We evaluated the linear agreement between PPCT and the two neurologic scales of PD severity (the UPDRS and the Hoehn and Yahr scales) using the Pearson correlation coefficient. A p value of less than 0.05 was considered statistically significant.

## Results

### Teaching population evaluation to establish peripapillary choroidal zones

The teaching population comprised 40 right eyes from healthy subjects and was used to identify 5 choroidal zones. The mean age of this population was 69.0 ± 7.9 years (range, 62–84 years). Of the 51 subjects, 14 (35%) were women. Mean spherical equivalent was 1.10 ± 1.35 D. Study zones were previously set regarding the thickness of the cubes for each acquisition point. Zone 1 corresponded to the optic nerve head area, and thus was not measured by the OCT and not incorporated into the study. Zone 2 included the cubes with a choroidal thickness of less than 105 μm, zone 3 included cubes with a thickness of 105 to 139 μm, zone 4 included cubes with a thickness of 140 to 174 μm, and zone 5 included cubes with a thickness of 175 μm or greater (Fig 1).

**Fig 2:**
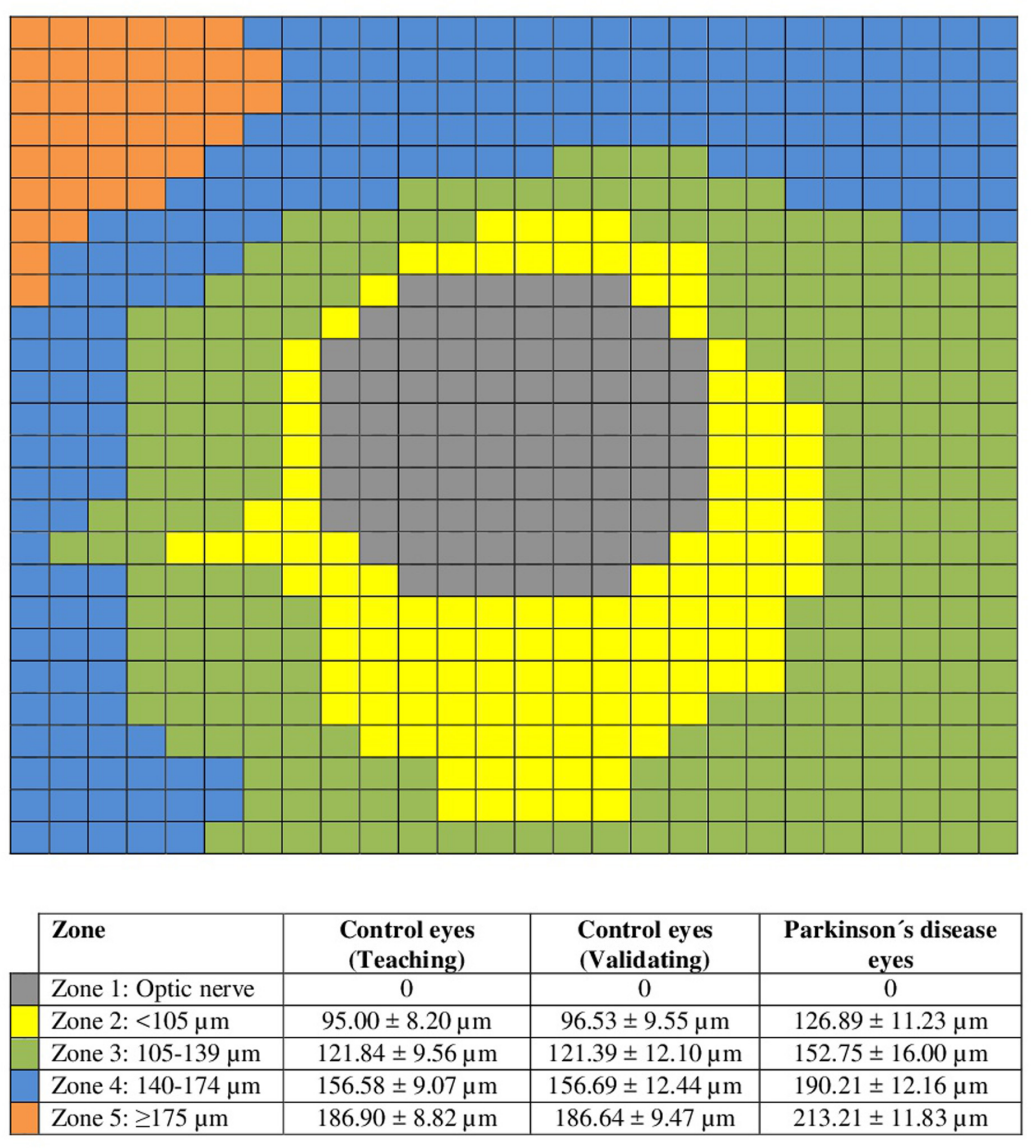
Schematic representation of the 5 zones defined in the 26×26 cube-grid using the peripapillary choroidal thickness measurements in the teaching control population: Zone 1, corresponding with optic nerve head in grey; zone 2 corresponds with mean peripapillary choroidal thickness (PPCT) <105 μm and is represented in yellow; zone 3 corresponds with mean PPCT ranging from 105 to 139 μm, and is represented in green; zone 4 corresponds with mean PPCT ranging from 140 to 174 μm, and is represented in blue; and zone 5 corresponds with mean PPCT ≥175 μm and is represented in orange. Bottom table: Mean PPCT ± standard deviation of each zone for the three groups evaluated (teaching control group, validating control group, and Parkinson´s disease group).

The mean choroidal thickness in zone 2 was 95.00 ± 8.20 μm and included 120 cubes of the choroidal grid; in zone 3 the mean thickness was 121.84 ± 9.56 μm and included 248 cubes; in zone 4 the mean thickness was 156.58 ± 9.07 μm and included 181 cubes; and in zone 5 the mean thickness was 186.90 ± 8.82 μm and included 31 cubes (Fig 2).

**Fig 3:**
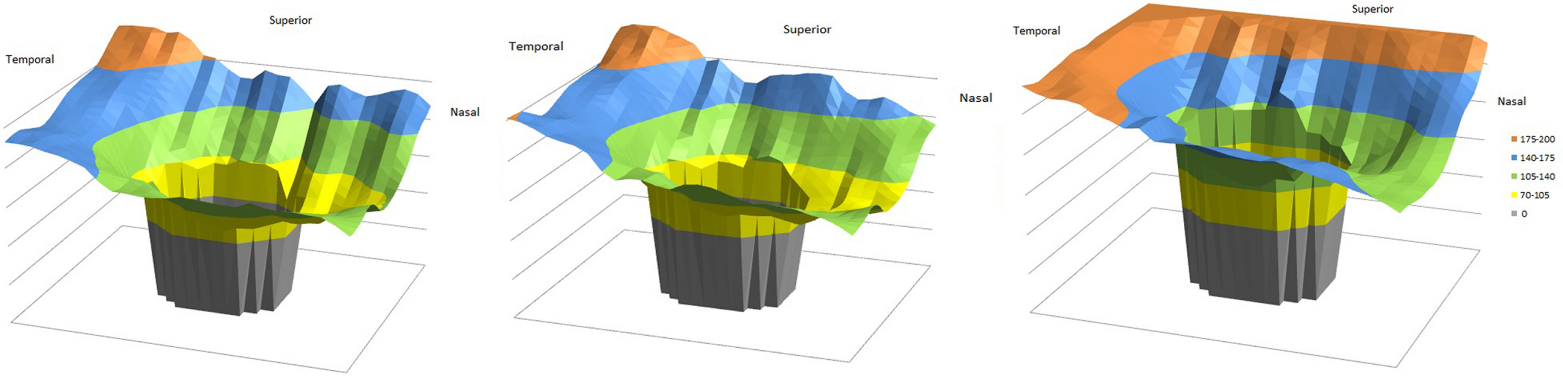
Three-dimensional graphical representation of the peripapillary choroidal thickness (PPCT) measurements in controls eyes (teaching population in left fig, validating population in middle fig) and Parkinson´s disease (PD) eyes in right fig. Grey, cubes corresponding with the optic nerve head; yellow, PPCT <105 μm; green, mean PPCT ranging from 105 to 139 μm; blue, mean PPCT ranging from 140 to 174 μm; and orange, mean PPCT ≥175 μm.

**Fig 4:**
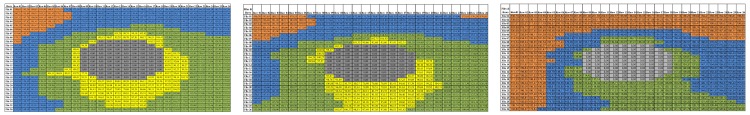
Representation of the mean peripapillary choroidal thickness (PPCT) for the 26×26 cube-grid centered on the optic disc for the three groups: the 40 right healthy eyes of the teaching population (left fig), the 40 right eyes of the healthy validating population (middle fig), and the 40 right eyes of the Parkinson´s disease (PD) patient group (right fig). Grey, cubes corresponding with the optic nerve head; yellow, mean PPCT <105 μm; green, mean PPCT ranging from 105 to 139 μm; blue, mean PPCT ranging from 140 to 174 μm; and orange, mean PPCT ≥175 μm. The temporosuperior choroid is the thickest, followed by superior, temporal, nasal, and inferior choroid.

Figs 3 and 4 show the five zones in the teaching population of healthy controls, that are roughly concentric to the optic nerve head, zone 2 mainly (the thinnest of the study zones with a minimum mean PPCT of 78 μm) located nearest the optic nerve head and inferior peripapillary choroid, zone 3 was mainly located in the inferior and nasal peripapillary choroid, zone 4 (around zone 3, especially in the temporal and superonasal areas), and zone 5 (the zone with the maximum mean PPCT of 205 μm, corresponding to the farthest cubes, mainly located in the superior and temporal peripapillary choroid).

### Validating population and statistical comparison between healthy and PD eyes

Once the study zones were established, a statistical comparison between control and PD eyes was performed on a different population of control eyes. A total of 40 right eyes from healthy subjects (independent from those subjects used in the teaching population) and 40 right eyes from PD patients were included in the study. The mean age of the healthy control group was 68.58 ± 7.17 years (range: 61 to 85 years) and the mean age of the PD group was 69.76 ± 6.45 years (range: 62 to 85 years). Of the 40 subjects in each group, 14 (35%) were women. Mean spherical equivalent was 0.13 ± 1.88 D in the control group (range, -2.50 to 2.50) and 0.14 ± 1.76 D in the PD group (range, -2.75 to 2.50). Age, sex, and spherical equivalent did not differ significantly between groups (p<0.05). The study population characteristics are summarized in Table 1.

**Table 1:** Descriptive, clinical data, choroidal thicknesses and volume for eyes of patients with Parkinson’s disease and from healthy controls (validating population).

	PD PATIENTS	CONTROLS	P*
Number of eyes	40	40	-
Age (years)	69.8 ± 6.5	68.6 ± 7.2	0.137
Sex (men/women)	26/14	26/14	0.901
Spherical Equivalent (D)	0.14 ± 1.76	0.13 ± 1.88	0.388
Number of myopic subjects (%) **	18 (45%)	17 4(42.5%)	0.404
Number of hyperopic subjects (%)**	6 (15%)	6 (15%)	0.945
BCVA (LogMAR)	0.11 ± 0.17	0.03 ± 0.16	0.005
IOP (mm Hg)	16.5 ± 2.0	16.2 ± 2.8	0.439
Disc area (mm^2^)	1.92 ± 0.37	1.89 ± 0.37	0.309
Unified Parkinson Disease Rating Scale	25.1 ± 5.4	-	-
Hoehn and Yahr Scale	2.7 ± 0.8	-	
Disease duration (years)	6.7 ± 2.3	-	-
Choroidal thickness (μm)			
Zone 2	126.89 ± 11.23	96.53 ± 9.55	<0.0001
Zone 3	152.75 ± 16.00	121.39 ± 12.10	<0.0001
Zone 4	190.21 ± 12.16	156.69 ± 12.44	<0.0001
Zone 5	213.21 ± 11.83	186.64 ± 9.47	<0.0001
Total	163.01 ± 29.90	131.51 ± 30.22	<0.0001
Number of peripapillary cubes			
70–104 μm thick	0	116	<0.0001
105–139 μm thick	165	258	<0.0001
140–174 μm thick	202	175	<0.0010
≥175 μm thick	221	39	<0.0001

Comparison of the choroidal thicknesses in the four established zones in the control group and Parkinson’s disease patients, and comparison between groups. Comparison of the number of peripapillary cubes with a thickness from 70 to 104 μm (zone 2), from 105 to 139 μm (zone 3); from 140 to 174 μm (zone 4), and ≥ 175 μm (zone 5). Zone 1 corresponds to the optic nerve head and was not included in the analysis.

* p: level of statistical significance in comparison between the two groups using Student’s t-test (except for sex, chi-square test). Data are mean ± standard deviation. Bold text indicates statistically significant results (p<0.05).

** Subjects were considered myopic when spherical equivalent was < -1D. Subjects were considered hyperopic when spherical equivalent was >1D. Abbreviations: PD, Parkinson´s disease; D, diopters; BCVA, best-corrected visual acuity; IOP, intraocular pressure.

“Drugs that enhance dopamine levels” was the most prescribed category (87% of patients) and combination therapy with levodopa and carbidopa was the most frequent treatment (46%). Sixty-five percent of treatments were categorized as “dopaminergic”, most of which were used in combination with the drugs included in the “Drugs that enhance dopamine levels” category. A small percentage of patients (8%) were prescribed drugs with no dopaminergic effects.

Comparison of the PPCT between the healthy controls and the PD eyes in the five zones described revealed a statistically thicker choroidal layer in all zones in the PD group (p<0.0001; Table 1 and Fig 2). The choroid followed a similar pattern in controls and PD; it was thicker in the superotemporal region, followed by the temporal, nasal, and inferior regions, as shown Figs 3 and 4. PPCT was higher in PD patients. The choroidal thicknesses did not correlate with disease severity.

### Evaluation of choroidal thicknesses in classic tomographic areas

The parapapillary choroidal thicknesses of the quadrants and sectors provided by the OCT analysis were also compared between PD patients and healthy controls, and revealed a significant increase in patients (Table 2).

**Table 2:** Macular and peripapillary choroidal thickness and volume measured with swept-source deep range imaging optical coherence tomography in patients with Parkinson’s disease and healthy controls.

**Peripapillary choroidal thickness (μm)**	**PD PATIENTS** **Mean (SD)**	**CONTROLS** **Mean (SD)**	**p**
Total	153.51 (61.32)	125.01 (54.76)	0.009
*Quadrants (x4)*			
Superior	163.01 (64.44)	146.65 (59.42)	0.121
Nasal	151.97 (52.21)	131.60 (55.05)	0.017
Inferior	127.11 (61.49)	100.23 (56.40)	0.007
Temporal	163.80 (75.96)	130.12 (53.58)	0.002*
*Sectors (x6)*			
Superotemporal	164.11 (65.00)	142.98 (56.81)	0.101
Superonasal	168.87 (64.05)	150.28 (64.41)	0.254
Nasal	149.11 (52.76)	128.82 (56.04)	0.023
Inferotemporal	129.71 (69.92)	94.15 (50.01)	0.002*
Inferonasal	126.01 (57.13)	100.02 (58.93)	0.015
Temporal	164.04 (77.92)	130.00 (56.21)	0.002*
**Peripapillary choroidal volume (mm^3^)**			
Total	3.61 (61.32)	2.94 (1.29)	0.007
*Quadrants (x4)*			
Superior	0.96 (0.38)	0.86 (0.35)	0.098
Nasal	0.89 (0.31)	0.77 (0.32)	0.012
Inferior	0.74 (0.36)	0.59 (0.33)	0.005
Temporal	0.96 (0.44)	0.76 (0.31)	0.001*
*Sectors (x6)*			
Superotemporal	0.64 (0.25)	0.56 (0.22)	0.081
Superonasal	0.66 (0.25)	0.59 (0.25)	0.178
Nasal	0.581 (0.21)	0.50 (0.22)	0.019
Inferotemporal	0.51 (0.27)	0.37 (0.20)	0.002*
Inferonasal	0.49 (0.22)	0.39 (0.23)	0.016
Temporal	0.64 (0.31)	0.51 (0.221)	0.001*

Bold letters indicate p<0.05. Asterisks mark significant values according to Bonferroni’s correction for multiple comparisons (p<0.005). Abbreviations: PD, Parkinson’s disease.

## Discussion

The involvement of the choroid in PD is unknown. To our knowledge, Eraslan et al.[25] published the only study that discusses the choroid in relation to PD and suggested that choroidal thickness is significantly diminished in PD patients [25]. In that study, a smaller sample size was evaluated and the measurements of the choroidal plexus were obtained using SD-OCT EDI. In SD-OCT EDI, the inner and outer borders of the choroid are determined manually by the observer [23], and the error in the thickness measurements must be estimated from the obtained images. In the present study, we used SS-OCT, in which the tunable laser source has a lower signal decay versus depth than previous SD-OCT devices. Thus, the chorioscleral border is more clearly delineated due to the reduction in speckle noise, providing more accurate manual and automated three-dimensional images of the retina and choroid [26]. In addition, with the SS-OCT device, observers only analyze a circle scan (SS-OCT only evaluates 12 central degrees around the optic nerve and reports manual measurements of the choroidal thickness for various points within this small area) [12], while the DRI-OCT used in our study provides a total of 588 automatic measurements of the choroidal thickness, with each measurement covering a broader area around the optic nerve (5.2x5.2 mm, corresponding to approximately 20 central degrees). Our present results revealed a significantly thickened choroid in the peripapillary area, which is opposite to the findings for the retina in PD and to the Eraslan et al. results [25].

RNFL thickness measurements by OCT are considered a useful indirect marker of the progression of brain atrophy in patients with PD [6,27]. Previous studies demonstrated macular thinning of all areas in PD patients compared with controls, [5,6], an inverse correlation with Hoehn and Yahr and UPDRS severity, and a positive correlation with the Schwab-England Activities of Daily Living scale [27]. Therefore, increased neurologic effects and severity of PD progression are linked to thinning of macular tissue [27]. Typical ophthalmic findings in PD patients are optic nerve atrophy and peripapillary RNFL thinning [6,9], but the secondary impact on other deeper ocular layers, such as the choroid, have not been evaluated.

In our study, PD patients showed a 31.5% increase in the choroidal thickness in zone 2, 25.8% increase in zone 3, 21.5% increase in zone 4, and 14.2% increase in zone 5 compared with controls. Therefore, the choroidal thickness in PD patients increased with increasing distance from the optic nerve head. The cause of this mean total increase of 24.0% in the choroidal thickness of PD patients is unknown. We found that all quadrants and peripapillary sectors provided by classic topographic OCT analysis were increased in PD compared with healthy controls. The choroidal thickening in PD was generalized, and affected all the topographic zones around the optical nerve.

A vascular-based influence for PD progression was recently proposed without clear results [28–30]. Despite the observation of vascular changes in vascular parkinsonism [28], no differences are observed in white matter vascularization in idiopathic PD compared to controls [29]. In a recent study, an increased density of string vessels (vessels with collapsed basement membranes, without endothelium, and with no functional circulation) was observed in the brain of PD patients, associated with neuronal loss [30]. Vascular ultrastructural alterations in the choroid of PD patients have not yet been described, thus we postulate that the increased choroidal thickness may be due to alterations in the perivascular connective tissue density despite the previously suggested hypoperfusion in these patients [25]. Also, SS-OCT technology provides increased depth analysis of the choroid compared to SD-OCT devices, and that also may account for the increased choroidal thickness compared to previous studies. More studies analyzing the macular and peripapillary choroid in PD using new SS-OCT technology and histologic analysis are needed to corroborate our findings.

The choroid is a dynamic structure and its thickness depends on several factors. For example, older age [31,32], higher IOP [33], higher myopia, and longer axial length [34,35] are associated with a thinner choroid. In the present study, however, we excluded subjects with high IOP, high myopia, and longer axial length. Several factors contribute to the conflicting findings in PPCT with OCT; such as different measurement techniques (most of them performed manually), and the dynamic and variable nature of the choroid [36]. New technologies, such as DRI SS OCT, provide not only better delineation of the retinal layers but also a much improved method for measuring a very large number of points in the peripapillary region and other regions farther from the choroid. With these tools, we can determine the true shape of the choroidal layer and possible differences due to various diseases.

Our intention was to first better understand the pattern of PPCT in normal eyes, establish different zones based on the distribution of choroidal thickness, and to compare these areas with eyes of patients with PD. Some histologic studies and others with SD-OCT demonstrated that the choroid tends to be thinner around the optic nerve head compared with the subfoveal choroid [32,35,37], which is consistent with our results. We found that the peripapillary choroid follows a pattern in healthy subjects and PD patients; and is thicker superiorly and thinner near the optic nerve head, especially inferiorly, which is consistent with other studies [38]. The choroidal pattern is the same in PD patients, but with thicker measurements. Our findings revealed that the peripapillary choroid was thicker in all zones in PD patients, and the differences between healthy and PD eyes tended to decrease with increasing distance from the optic nerve head.

This study included a teaching population to determine the pattern of the topographic distribution of thickness in the peripapillary choroidal layer, and a validating population to compare the thicknesses of the PD patients with an independent sample of healthy controls. Our research team, as well as others, use this method of utilizing teaching and validating populations to avoid the overestimation effect caused by using the same subjects to create the model and to compare that model with controls [39,40].

This study has several limitations. First, we did not collect data on axial length; however, we demonstrated that there was no significant difference in refractive error among groups. The axial length and refractive error variables are related, but not interchangeable, because axial length tends to not change after the second decade of life, except in cases with some pathology. Instead, the refractive error may fluctuate or change due to different factors [24]. The findings from a recent study by Ikuno et al. [41] using OCT, however, suggest that refractive error and axial length may have a similar relationship with OCT measurements. Second, the choroid can be influenced by circadian changes [42], and we did not perform OCT examinations at the exact same time of day in all participants; although all of the OCT studies were acquired in the evening (between 16.00 and 20.00). Third, we only measured choroidal thickness in the right eyes of each participant, and inter-eye differences were not assessed. Fourth, the presence of motor symptoms in some of the patients may have affected the masking (i.e., masking was more effective in patients with mild or non-evident symptoms). Finally, we did not establish the reliability of automated choroidal thickness measurements with this new SS-OCT. It would be interesting to investigate the accuracy of this device in another study.

Imaging techniques such as OCT are noninvasive, inexpensive, and fast, and may be useful for monitoring treatment efficacy, detecting progressive neurodegeneration, and improving diagnostic procedures in neurologic diseases (such as making a differential diagnosis between PD and essential tremor) [43]. In our study, we did not find that choroidal thickness was correlated with PD severity, but studies with a larger sample size may find significant associations. A subgroup analysis according to PD medication was not possible as most of our patients were prescribed combined treatment and the sample size per subgroup was not large enough to compare the subgroups. More studies are needed to corroborate these results and to evaluate the usefulness of choroidal measurements in PD and the role of this evaluation in clinical practice.
